# Chronic low-dose Δ^9^-tetrahydrocannabinol (THC) treatment stabilizes dendritic spines in 18-month-old mice

**DOI:** 10.1038/s41598-022-27146-2

**Published:** 2023-01-25

**Authors:** Joanna Agnieszka Komorowska-Müller, Anne-Kathrin Gellner, Kishore Aravind Ravichandran, Andras Bilkei-Gorzo, Andreas Zimmer, Valentin Stein

**Affiliations:** 1 Medical Faculty, Institute of Molecular Psychiatry, University of Bonn, Bonn, Germany; 2 Medical Faculty, Institute of Physiology II, University of Bonn, Bonn, Germany; 3 Department of Psychiatry and Psychotherapy, University Hospital Bonn, Bonn, Germany

**Keywords:** Cell biology, Molecular biology, Neuroscience

## Abstract

Cognitive functions decline during aging. This decline could be caused by changes in dendritic spine stability and altered spine dynamics. Previously, we have shown that a low dose chronic THC treatment improves learning abilities in old whereas impairs learning abilities in young mice. The mechanism underlying this age-dependent effect is not known. Dendritic spine stability is a key for memory formation, therefore we hypothesized that THC affects spine dynamics in an age-dependent manner. We applied longitudinal 2-photon in vivo imaging to 3- and 18-month-old mice treated with 3 mg/kg/day of THC for 28 days via an osmotic pump. We imaged the same dendritic segments before, during and after the treatment and assessed changes in spine density and stability. We now show that in old mice THC improved spine stability resulting in a long-lasting increase in spine density. In contrast, in young mice THC transiently increased spine turnover and destabilized the spines.

## Introduction

The endocannabinoid tone changes during the course of aging: endocannabinoid signaling is high during adolescence and declines in aged rodents^1–3^. This change in endocannabinoid tone includes a decrease of endocannabinoid levels, brain area-dependent alterations of cannabinoid receptors 1 (CB_1_Rs) expression and alterations in their coupling to G-proteins. Moreover, mice lacking CB_1_R exhibited an accelerated aging phenotype with premature cognitive decline, gliosis and increased expression of inflammatory cytokines in the brain^4–6^. Whereas, rising the endocannabinoid tone using exogenous ligands of cannabinoid receptors gave promising results in counteracting age-related changes^7,8^.

We demonstrated earlier that increasing the endocannabinoid tone in old mice through continuous administration of a low dose (3 mg/kg/day) Δ^9^-tetrahydrocannabinol (THC) over 28 days counteracted the age-related decline in cognitive performance and age-induced synaptic loss. However, the same treatment had opposite effects on the cognitive performance of young mice^9^. This effect was dependent on the CB_1_R of the forebrain glutamatergic-cells^9^. As cognitive performance depends on the plasticity of synapses and spine dynamics of glutamatergic neurons, which is altered during the aging process^10–12^, we hypothesize now that supplementing aged animals with a low dose of THC could reestablish the spine dynamics of young mice in old mice.

### THC treatment long-lastingly increased spine density in 18-month-old, but not in 3-month-old mice

**Figure 1:**
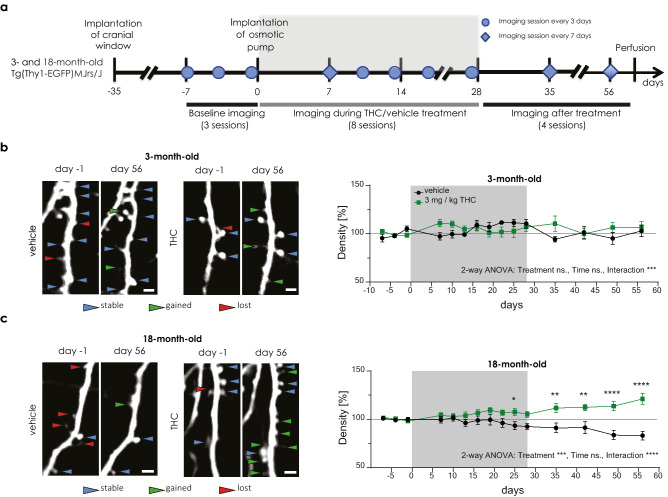
Long-term low-dose THC treatment increases spine density in old, but not in young mice. (**a**) Time line of the 2-photon imaging experiment. Representative images of the same dendritic segment at day -1 and day 56 acquired during in vivo imaging from 3- (**b**) and 18- (**c**) month-old mice treated with vehicle or THC. Scale bar is 2 µm. Spine density changes in 3- (**b**) and 18-month-old mice (**c**). Spine density was normalized to the mean spine density during baseline imaging. Grey box indicates the treatment duration. 3-month-old mice (THC n = 12 ROIs, N = 4 mice; vehicle n = 10 ROIs, N = 4 mice); interaction effect F_14,192_ = 3.247, *p* = 0.0001; 18-month-old mice (THC n = 22 ROIs, N = 10 mice; vehicle group n = 17 ROIs, N = 8 mice) treatment effect F_1,37_ = 13.04, *p* = 0.0009; interaction effect F_14,396_ = 5.069, *p* < 0.0001 (day 25 *p* = 0.0141; day 35 *p* = 0.0036; day 42 *p* = 0.0035; day 48 and 56 *p* < 0.0001). THC effect in comparison to day -1 (day 35 *p* = 0.0093, day 42 *p* = 0.0008, day 49 *p* = 0.0064, day 56 *p* = 0.0007); vehicle effect in comparison to day -1 (day 56 *p* = 0.0322) (not shown on the graph). Error bars indicate mean ± SEM; 2-way ANOVA (Mixed-effects analysis) followed by Sidak’s multiple comparison test; * *P* < 0.05, ** *P* < 0.01; *** *P* < 0.001; **** *P* < 0.0001. ns. - not significant.

To test this idea, we repeatedly imaged dendritic segments of layer V pyramidal neurons of the somatosensory cortex in 3- and 18-month-old GFP-M male mice through a chronic cranial window. The imaging started 1 week before THC treatment, lasted throughout the 28-day THC treatment and ended 28 days after THC treatment had terminated (Fig. 1a, Extended Fig. 1). Consistent with previous studies^10,11^, old mice showed increased spine density, higher spine turnover and decreased spine stability compared to young mice during baseline conditions (Extended Fig. 2). Notably, THC treatment for 28 days had no effect on spine density in young mice (Fig. 1b), but spine density increased in old mice in comparison to the vehicle-treated mice (Fig. 1c). This effect became significant at day 25 of the treatment and manifested throughout the entire post-treatment observation period (Fig. 1c). Correspondingly, THC significantly increased spine density at days 35, 42, 49 and 56 of the treatment in comparison to baseline day -1. Whereas, spine density decreased in the vehicle treated group at day 56 (post-hoc results not shown in the graph).

**Figure 2:**
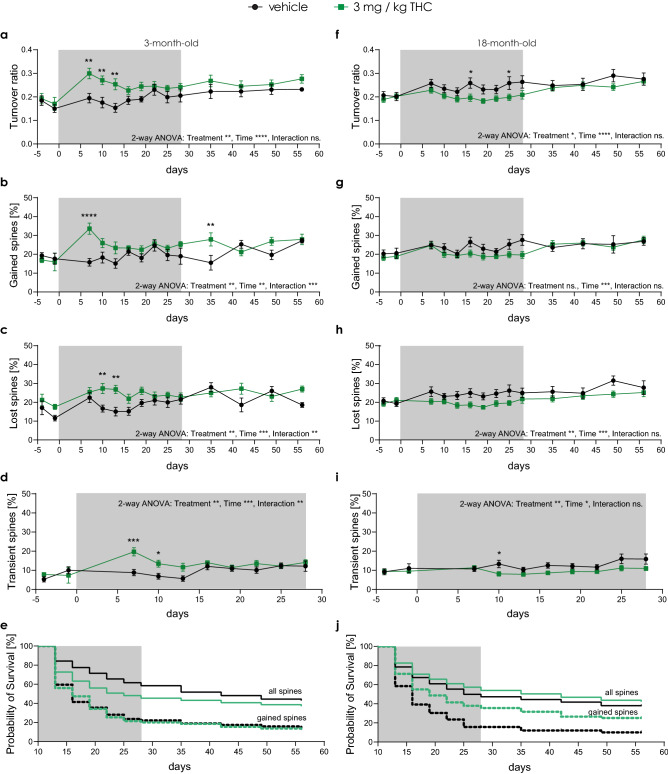
Long-term low-dose THC treatment differently alters spine dynamics in old and young mice. (**a, f**) Turnover ratio—number of lost and gained spines between two consecutive time points divided by the total number of spines in both time points. Percentage of spines that were gained (**b, g**) and lost (**c, h**) between two consecutive time points. Percentage of spines that were transient (**d, i**)—gained in one time point and lost in the consecutive one. (**e, j**) Survival probability of spines that were gained between day 7 and day 10 (dashed line) of the treatment and all spines present at day 10 (full line). Grey box indicates the treatment duration. (**a—e**) 3-month-old mice (THC n = 12 ROIs, N = 4 mice; vehicle n = 10 ROIs, N = 4 mice); (**a**) turnover ratio: time effect F_13,179_ = 3.718, *p* < 0.0001; treatment effect F_1,20_ = 10.93, *p* = 0.0035 (day 7 *p* = 0.0017; day 10 *p* = 0.0033; day 13 *p* = 0.0014); (**b**) gained spines: time effect F_13,179_ = 2.291, *p* = 0.0080; treatment effect F_1,20_ = 9.589, *p* = 0.0057; interaction effect F_13,179_ = 3.050, *p* = 0.0004 (day 7 *p* < 0.0001; day 35 *p* = 0.0031); (**c**) lost spines: time effect F_13,179_ = 3.245, *p* = 0.0002; treatment effect F_1,20_ = 10.15, *p* = 0.0046; interaction effect F_13,179_ = 2.382, *p* = 0.0057 (day 10 *p* = 0.0054; day 13 *p* = 0.0011); (**d**) transient spines: time effect F_9,117_ = 3.715, *p* = 0.0004; treatment effect F_1,20_ = 9.299, *p* = 0.0063; interaction effect F_9,117_ = 2.812, *p* = 0.0050 (day 7 *p* = 0.0002; day 10 *p* = 0.0237); (**e**) survival all spines: *p* = 0.0002; (**f–j**) 18-month-old mice (THC n = 22 ROIs, N = 10 mice; vehicle group n = 17 ROIs, N = 8 mice); (**f**) turnover ratio: time effect F_13,339_ = 4.759, *p* < 0.0001 ; treatment effect F_1,36_ = 5.403, *p* = 0.0259 (day 16 *p* = 0.0424; day 25 *p* = 0.0448); (**g**) gained spines: time effect F_13,339_ = 3.208, *p* = 0.0001; (**h**) lost spines: time effect F_13,339_ = 2.356, *p* = 0.0050; treatment effect F_1,36_ = 7.416, *p* = 0.0099; (**i**) transient spines: time effect F_9,239_ = 1.948, *p* = 0.0462; treatment effect F_1,36_ = 8.564, *p* = 0.0059 (day 10 *p* = 0.0343); (**j**) survival gained spines: *p* < 0.0001; survival all spines: *p* = 0.0123. Each data point represents one ROI. Error bars indicate mean ± SEM; 2-way ANOVA (Mixed-effects analysis) followed by Sidak’s multiple comparison test; for survival probability analysis (e and j) Kaplan–Meier plot and log rank test (Mantel-Cox test) were used; * *P* < 0.05, ** *P* < 0.01; *** *P* < 0.001; **** *P* < 0.0001. ns. - not significant.

### THC treatment altered spine dynamics differently in 3- and 18-month-old mice

Although spine density was not altered in young mice during and after THC treatment, spine dynamics showed a transient alteration. Between day 7 and 13 of the treatment, the spine turnover ratio was increased (Fig. 2a), with a rise in spine formation at day 7 and and a subsequent rapid loss of most of the recently gained spines (Fig. 2 b and c). This consequently led to an increased rate of transient, unstable spines (Fig. 2d), also reflected by a significant reduction of the probability of spine survival (Fig. 2e). In contrast, in old mice, no change in spine gain (Fig. 2g), but a reduction in spine loss (Fig. 2h) led to a lowered turnover ratio (Fig. 2f) and decreased the number of transient spines (Fig. 2i). Together, this resulted in an elevated probability of spine survival underlying the increasing spine density (Fig. 2j). Moreover, opposite to the young mice, in old mice changes were smaller in magnitude, but long-lasting, starting from around day 13–16 into the treatment. Thus, cumulatively resulting in observed elevated spine density.

### THC treatment in 18-month-old mice counteracted age-related changes on the level of spine dynamics

**Figure 3:**
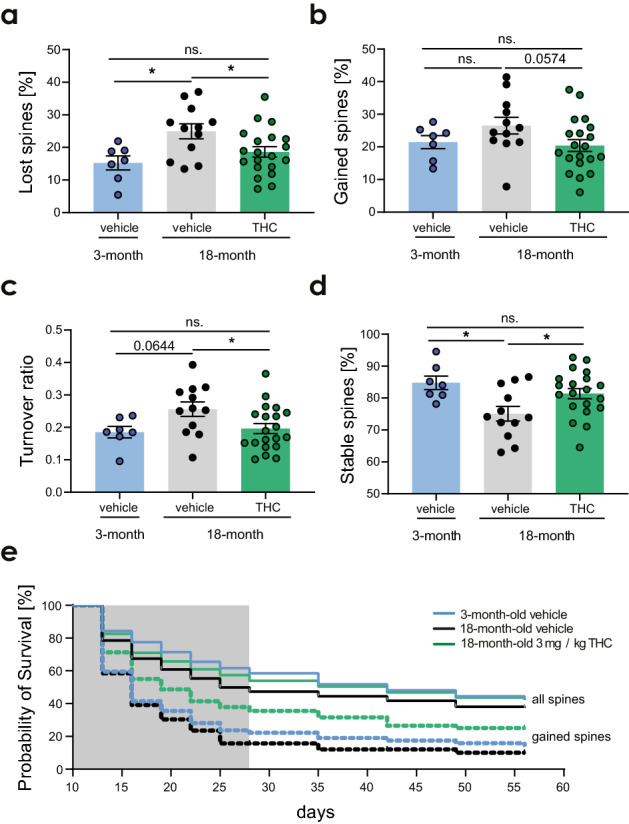
THC counteracts the effects of aging on the level of spine dynamics. Percentage of spines that were lost (**a**) or gained (**b**). (**c**) Turnover ratio—number of lost and gained spines between two consecutive time points divided by the total number of spines in both time points. (**d**) Percentage of spines that were stable between two consecutive time points. Spine dynamics from day 13 to day 16 of the treatment. For 3-month-old mice (n = 7 ROIs), for 18-month-old mice (n = 12–20 ROIs / treatment). Each data point represents one ROI. Data was analyzed using one-way ANOVA and Dunnett’s multiple comparison test for comparison of 3-months old vehicle group with the 18-month-old vehicle and THC-treated groups. Differences between the 18-month-old vehicle and THC groups were detected using unpaired t-test. (**a**) Group effect F_2,36_ = 4.755, *p* = 0.0147 (3- vs. 18-month-old vehicle *p* = 0.0129); 18-month-old vehicle vs. THC t_30_ = 2.321, *p* = 0.0273; (**b**) group effect F_2,36_ = 2.244, *p* = 0.1207; (**c**) group effect F_2,36_ = 3.462, *p* = 0.0422 (3- vs. 18-month-old vehicle *p* = 0.0644); 18-month-old vehicle vs. THC t_30_ = 2.241, *p* = 0.0326 (**d**) group effect F_2,36_ = 4.755, *p* = 0.0147 (3- vs. 18-month-old vehicle *p* = 0.0129); 18-month-old vehicle versus THC t_30_ = 2.321, *p* = 0.0273. (**e**) Survival probability of spines that were gained between day 7 and day 10 (dashed line) of the treatment and all spines that were present at day 10 (full line). THC treatment elevated spine survival of all spines in old mice to the level of young vehicle-treated mice; while spine survival in vehicle treated old mice was significantly reduced (all spines 3- vs. 18-months-old vehicle groups: *p* = 0.0023). THC-treatment also increased the survival of gained spines notably in comparison to the 3-months-old vehicle group (3-month-old vehicle group vs. 18-month old THC group: *p* = 0.0076). For survival probability analysis Kaplan–Meier plot and log rank test (Mantel-Cox test) were used. Grey box indicates the treatment duration. Error bars indicate mean ± SEM; * *P* < 0.05, ** *P* < 0.01, *** *P* < 0.001. ns. - not significant.

Next, we sought to further dissect the underlying effects of the increase in spine density in THC-treated old animals. As our previous observations showed that THC-caused improvement of spatial memory in old mice as early as treatment day 14^9^, we compared the spine dynamics specifically on day 16 of THC treatment. At day 16, THC-treated old mice compared to vehicle-treated old mice showed a decreased spine loss (Fig. 3a), but unaltered spine gain (Fig. 3b), resulting in a reduced spine turnover (Fig. 3c). Together, this led to an increased spine stability in THC-treated 18-month-old mice (Fig. 3d). These findings are consistent with the timing of the THC-induced cognitive improvement in 18-month-old mice in our previous work^9^.

The counteracting effect of THC on the age-related change in spine dynamics was further revealed when we compared spine dynamics of 3- and 18-month-old vehicle treated to 18-month-old THC-treated mice (Fig. 3a–d). The comparison of 3- to 18-month-old vehicle treated mice revealed a decreased spine stability (Fig. 3d) resulting from increased loss of spines (Fig. 3a). Spine loss and spine stability did not differ significantly between 3-month-old vehicle and 18-month-old THC groups.

Thus, the general age effect on spines was absent in the THC-treated 18-month-old mice. These findings further support that THC counter-acted aging on the level of spine dynamics.

Finally, we looked at the spine stability over time and compared the probability of spine survival between old and young mice either of all spines present at day 10 or those spines that formed between day 7 and day 10 during the THC treatment. THC treatment elevated probability of spine survival of all spines in old mice to the level of young vehicle-treated mice, while probability of spine survival in vehicle treated old mice was significantly reduced. A similar effect was observed for gained spines, which survived relevantly longer after THC in the old group. In summary, THC treatment restored spine survival to the level of young mice or even exceeded it in case of spines that were formed during the THC treatment itself (Fig. 3e).

## Discussion

Several studies have shown that spine stabilization is a key step for long-term consolidation and storage of new information and skills^13–15^, although the underlying mechanisms have not been entirely elucidated. Importantly, it has been noted that spine stability changes during the aging process^10,11,16,17^ possibly contributing to age-related learning deficits. Our results are consistent with these findings and suggest a critical role for endocannabinoid signaling in the modulation of age-related changes in synaptic stability. We have demonstrated that a low dose of THC leads in old mice to a significant change in spine dynamics that resembles the situation in young mice. Furthermore, the THC-induced increase in spine density lasted at least for 4 weeks following THC treatment. This correlates with our previously reported improved spatial memory performance in old mice^9^. In contrast, in young mice THC only caused a transient increase in spine turnover and spine destabilization, which correlates with decreased memory performance in young mice after THC treatment^9^. It has been shown that increased spine density positively correlates with cognitive performance in mice^18^. Reversely, dendritic spine loss, especially of thin spines, correlated with memory impairment in Alzheimer’s Disease patients and preceded neuronal loss^19–21^. These studies had investigated a broad range of neocortical and hippocampal areas. We have targeted the somatosensory cortex as the CB_1_R is present in most brain areas, but its density is particularly high in the cortex, hippocampus, and cerebellum^22–24^. The hippocampus is important for learning and memory, however, in vivo 2-photon imaging of the hippocampus requires removal or at least severe manipulation of the overlaying cortex. While the removal of the cortex has been done to study the hippocampus in vivo^25^, this method is controversially discussed since the removal of the cortex might impact brain function and behavior. Therefore, we chose to image the somatosensory cortex, also because it had been investigated previously for physiological spine dynamics in aging mice^10^. As the CB_1_R density is high in both hippocampus and somatosensory cortex, we speculate that the effect of the THC treatment on spine dynamics should be similar in both brain areas.

We administered a relatively low amount of THC (3 mg/kg body weight/day) continuously released by an osmotic pump which did not induce psychotropic effects characterized by catalepsy, analgesia, hypothermia, hypomotility, or tolerance development^26–28^. Hence, it might be possible that the effects of high doses of THC on spine dynamics might be different or even opposite to what we observed. For instance, a dose-dependent, biphasic effect of THC was shown for its anxiolytic/anxiogenic properties, but also in relation to cognition^29,30^. On the other hand, not only 3 mg/kg that we used in our study, but also lower doses seem to cause a similar effect in old mice. Recent investigations found improved spatial memory of old mice after 1 mg/kg/day chronic THC treatment^31^ and after an injection of 0.001 mg/kg of THC^32^.

We have observed that the THC treatment effect was age-dependent as THC caused a transient effect on spine dynamics in young mice and a long-lasting effect in old mice. This can be caused by tolerance development to THC as it was shown that in contrast to young mice, tolerance development to THC is impaired in aged mice^28^. However, tolerance development only explains the temporal nature of the effect we observed, but not the direction of it such as increase/decrease of spine stability and its consequences on cognitive performance. The direction of the THC effect, most likely, depends on the varying brain chemistry at different age-groups which includes age-related decline in baseline endocannabinoid system activity^1–3^.

The differential cellular and behavioral effects of THC in different age groups were also manifested in corresponding transcriptional changes, with THC-treated old mice showing similar transcriptional patterns as young control animals^9^. In old mice, THC decreased the methylation of H3K9 and upregulated brain-derived neurotrophic factor (BDNF) levels^9^, which has been associated with increased spine density and memory performance^33^. Moreover, activation of Tropomyosin receptor kinase B (TrkB), that BDNF binds to, was shown to stabilize dendritic spines and increase spine size^34,35^, which could potentially explain the spine density increase observed in our study. Alternatively, THC by activating CB_1_R could modulate the WAVE1 Complex and thus plays a role in structural plasticity via the regulation of actin polymerization^36^.

Taken together, 3 mg/kg/day chronic THC treatment has opposing effects on spine dynamics of young and old animals. In particular, THC treatment long-lastingly increased spine stability in the somatosensory cortex of old mice. This finding may have important medical implications providing a therapeutic option in age-related disorders with synaptic destabilization and loss.

## Experimental procedures

### Mice

Three-month-old and 18-month-old (16.5–19-month-old) Tg(Thy1-EGFP)MJrs/J (GFP-M) male mice^37^, IMSR Cat# JAX:007,788, RRID:IMSR_JAX:007,788) that express enhanced green fluorescent protein (eGFP) under the control of the Thy1 promoter resulting in a sparse neuronal labeling were used in this study. Mice were bred in the animal facility of the Medical Faculty of the University of Bonn. After weaning mice were group-housed (2–5 mice/cage) in standard laboratory cages with automatic ventilation system and ad libitum water and food access under a 12-h light–dark cycle. Care of the animals and conduction of the experiments followed the guidelines of the European Communities Directive 86/609/EEC and the German Animal Protection Law regulating animal research and were approved by the ethics committee of the Landesamt für Natur, Umwelt und Verbraucherschutz Nordrhein-Westfalen the LANUV NRW, Germany (81-02.04.2018.A027). All experiments were performed in accordance with relevant guidelines and regulations as well as were carried out in accordance with ARRIVE guidelines. In the 3-month-old group 8 mice were analyzed (4 vehicle, 4 treatment); in 18-month-old group 18 mice were analyzed (8 vehicle, 10 treatment). Four independent groups were tested. One group with 3-month-old and three groups with 16.5–19-month-old mice. Old groups were pooled together and treated as a single group for analysis.

### Cranial window implantation

Mice were anaesthetized with an intraperitoneal (i.p.) injection of medetomidin 0.5 mg/kg, midazolam 5 mg/kg, fentanyl 0.05 mg/kg body weight (BW). 5 mg/kg BW carprofen was injected s.c. for perioperative analgesia. Mouse breathing was checked throughout the surgery, body temperature was kept constant using a heating pad. Eye ointment was used to protect from eye dryness (Bepanthen). The mouse head was epilated and fixed in a stereotaxic frame. A circular trepanation 3–4 mm in diameter was carefully done over the left somatosensory cortex exposing the dura. The brain was covered with a 5 mm glass plate (Menzel) and sealed with cyanoacrylate (UHU) and dental cement (Paladur). A small Delrin bar with threaded holes was glued on the right hemisphere to fix the animal under the microscope. After surgery the anesthesia was reversed with atipamezol 2.5 mg/kg, flumazenil 0.5 mg/kg, naloxone 1.2 mg/kg BW. The mouse was placed under a warming lamp and monitored until fully awake. Every 12 h for 3 days, 0.1 mg/kg BW buprenorphine was applied s.c. for post-surgery analgesia. After the cranial window surgery, mice were single-housed. Animals had 3–4 weeks to recover before the chronic imaging was started.

### Drug treatment

Twenty eight-day releasing osmotic pumps (alzet; model 1004) were prepared according to the manufacturer's protocol to attain a 3 mg/kg/day dose with either THC or vehicle (ethanol:cremophor:saline, 1:1:18) solutions. Osmotic pumps were implanted subcutaneously on the back of the mice under anesthesia. Mice were distributed between the treatment group and the control group to ensure equal numbers and quality of dendritic segments to analyze. The experimenter was blinded for the treatment during all imaging sessions and spine analysis.

### 2-photon imaging

For imaging mice were sedated with an i.p. injection of medetomidin 0.5 mg/kg, midazolam 5 mg/kg BW and the head was fixed under the microscope. The body temperature was kept constant and the eyes were protected from dehydration with eye ointment (Bepanthen). We used a custom built 2-photon microscope driven with a Ti:sapphire Laser (Chameleon Vision-S, Coherent) running at 910 nm for GFP excitation. The setup was controlled by ScanImage (Vidrio Technologies) software^38^, running on MATLAB (Mathworks). 3–5 regions of interest (ROIs) were obtained from each mouse using a 40 × objective (NA 0.8, LumPlanFl, Olympus). Each ROI included multiple apical dendritic segments from layer V pyramidal neurons. To confirm that the dendritic segments belonged to layer II/III or V neurons, we followed dendritic segment towards the soma. Only ROIs in which spines were clearly visible were included into the analysis. To repeatedly find the same dendritic segments, blood vessels and the shape of neighboring dendrites were used as landmarks. In each time point from each imaging region one z-stack image was acquired of 1024 × 1024 pixels (0.065 μm/pixel) resolution and 16-bit depth using a 0.43 Hz frame rate resulting in a pixel dwell time of 2,000 ns. In the young group a step size of 0.51 µm was used, but in old animals it was changed to 0.8 µm to decrease the imaging time. Each frame was acquired 4 times and averaged to increase signal to noise ratio. Overview pictures were done during every imaging session to observe the stability of the window. After imaging, mice were woken up by an i.p. injection of atipamezol 2.5 mg/kg, flumazenil 0.5 mg/kg BW. Mice were placed under a warming lamp and monitored until fully awake.

### Dendritic spine analysis

Spine analysis was performed using a MATLAB script “Spine Analysis”^14^ provided with r3.8 ScanImage^38^. In general, 2–3 ROIs were analyzed per animal, in some cases we could only analyze 1 ROI (5 out of 26 mice) or even 4 ROIs (2 out of 26 mice). Only images that allowed for a visual identification of spines were used in the spine analysis. The number of ROIs and mice analyzed in each time point can be found in Supplementary Table 1. Image analysis consisted of two phases: annotation and correlation. First, each spine was manually annotated as a single line from the dendritic shaft to the tip of the protrusion. Only protrusions longer than 0.4 µm were considered as spines^14^. Next, annotated protrusions were correlated between time points. Protrusions could fall into one of the following categories: stable, gained, lost, and transient depending on their consecutive presence or absence (Fig. 1b). Spines were defined as stable: when the element was present on both consecutive time points; gained: when the element was not there on one time point but appeared on the consecutive one; lost: when the element was gone on the consecutive time point; transient: when gained on one time point and lost in the next one. Turnover ratio was calculated as the number of lost and gained spines between two time points divided by total number of spines in both time points. Within each ROI, we have measured the total length of each dendritic segment and quantified the number of present spines within the measured dendritic segment. To obtain the spine density we divided the sum of all annotated spines by the total length of all measured dendrites within a ROI. The reported spine dynamics were calculated using a custom written Python script (https://github.com/danielmk/spine_annotations_processing).

### Statistical analysis and data presentations

Microsoft Excel was used for data analysis followed by statistical analysis and data visualization in GraphPad Prism 8, GraphPad Software, San Diego, California USA, www.graphpad.com. For presentation, representative images were post-processed in Fiji. Figures were created in Adobe Illustrator CS5.1. For time-series experiments 2-way ANOVA (Mixed-effects model (REML)) was used followed by Sidak’s multiple comparison test. For survival probability analysis log-rank test (Mantel-Cox test) was used. Data sets with two independent groups were analyzed using unpaired t-test or U-test. Prior to this distribution was checked against normal distribution using the Anderson–Darling test. For analysis with three independent groups one-way ANOVA was applied after validation of normal distribution followed by Dunnet’s multiple comparison test. Statistical significance was stated when *p*-value < 0.05 at a 95% confidence interval with two-sided testing. *p*-values were reported in all figures as * *p* < 0.05, ** *p* < 0.01; *** *p* < 0.001; **** *p* < 0.0001. A detailed statistics table can be found as Supplementary Table 2.

## Supplementary Information

Supplementary Information.

## Data Availability

Datasets are available upon request from the corresponding authors—Valentin Stein and Andreas Zimmer. The raw data supporting the conclusions of this article will be made available by the authors, without undue reservation, to any qualified researcher.

## References

[1] Berrendero F (1998). Changes in cannabinoid receptor binding and mRNA levels in several brain regions of aged rats. Biochim. Biophys. Acta (BBA)-Mol. Basis Dis..

[2] Romero J (1998). Loss of cannabinoid receptor binding and messenger RNA levels and cannabinoid agonist-stimulated [35S] guanylyl-5′-O-(thio)-triphosphate binding in the basal ganglia of aged rats. Neuroscience.

[3] Piyanova A (2015). Age-related changes in the endocannabinoid system in the mouse hippocampus. Mech. Ageing Dev..

[4] Bilkei-Gorzo A (2005). Early age-related cognitive impairment in mice lacking cannabinoid CB1 receptors. Proc. Natl. Acad. Sci..

[5] Bilkei-Gorzo A (2012). Early onset of aging-like changes is restricted to cognitive abilities and skin structure in Cnr1-/- mice. Neurobiol. Aging.

[6] di Marzo V, Stella N, Zimmer A (2015). Endocannabinoid signalling and the deteriorating brain. Nat. Rev. Neurosci..

[7] Marchalant Y, Cerbai F, Brothers HM, Wenk GL (2008). Cannabinoid receptor stimulation is anti-inflammatory and improves memory in old rats. Neurobiol. Aging.

[8] Marchalant Y (2009). Cannabinoids attenuate the effects of aging upon neuroinflammation and neurogenesis. Neurobiol. Dis..

[9] Bilkei-Gorzo A (2017). A chronic low dose of Δ9-tetrahydrocannabinol (THC) restores cognitive function in old mice. Nat. Med..

[10] Mostany R (2013). Altered synaptic dynamics during normal brain aging. J. Neurosci..

[11] Davidson AM, Mejiá-Gómez H, Jacobowitz M, Mostany R (2020). Dendritic spine density and dynamics of layer 5 pyramidal neurons of the primary motor cortex are elevated with aging. Cereb. Cortex.

[12] Huang L, Zhou H, Chen K, Chen X, Yang G (2020). Learning-dependent dendritic spine plasticity is reduced in the aged mouse cortex. Front. Neural Circuits.

[13] Divecha N (2010). Lipid kinases: charging PtdIns(4,5)P2 synthesis. Curr. Biol..

[14] Holtmaat A (2009). Long-term, high-resolution imaging in the mouse neocortex through a chronic cranial window. Nat. Protoc..

[15] Yang G, Pan F, Gan WB (2009). Stably maintained dendritic spines are associated with lifelong memories. Nature.

[16] Bloss EB (2011). Evidence for reduced experience-dependent dendritic spine plasticity in the aging prefrontal cortex. J. Neurosci..

[17] Dickstein DL, Weaver CM, Luebke JI, Hof PR (2013). Dendritic spine changes associated with normal aging. Neuroscience.

[18] Mahmmoud RR (2015). Spatial and working memory is linked to spine density and mushroom spines. PLoS One.

[19] Boros BD (2017). Dendritic spines provide cognitive resilience against Alzheimer’s disease. Ann. Neurol..

[20] DeKosky ST, Scheff SW (1990). Synapse loss in frontal cortex biopsies in Alzheimer's disease: correlation with cognitive severity. Ann. Neurol..

[21] Terry RD, Masliah E, Salmon DP, Butters N, DeTeresa R, Hill R, Hansen LA, Katzman R (1991). Physical basis of cognitive alterations in alzheimer's disease: synapse loss is the major correlate of cognitive impairment. Ann. Neurol..

[22] Eggan SM, Lewis DA (2007). Immunocytochemical distribution of the cannabinoid CB1 receptor in the primate neocortex: a regional and laminar analysis. Cereb. Cortex.

[23] Herkenham M, Lynn AB, Little MD, Johnson MR, Melvin LS, de Costa BR, Rice KC (1990). Cannabinoid receptor localization in brain. Proc. Natl. Acad. Sci..

[24] Mechoulam R, Parker LA (2013). The endocannabinoid system and the brain. Ann. Rev. Psychol..

[25] Mizrahi A, Crowley JC, Shtoyerman E, Katz LC (2004). High-resolution in vivo imaging of hippocampal dendrites and spines. J. Neurosci..

[26] Moore CF, Weerts EM (2022). Cannabinoid tetrad effects of oral Δ9-tetrahydrocannabinol (THC) and cannabidiol (CBD) in male and female rats: sex, dose-effects and time course evaluations. Psychopharmacology.

[27] Metna-Laurent M, Mondésir M, Grel A, Vallée M, Piazza PV (2017). Cannabinoid-induced tetrad in mice. Curr. Protoc. Neurosci..

[28] Feliszek M (2016). Lack of hippocampal CB1 receptor desensitization by Δ9-tetrahydrocannabinol in aged mice and by low doses of JZL 184. Naunyn Schmiedebergs Arch. Pharmacol..

[29] Childs E, Lutz JA, de Wit H (2017). Dose-related effects of delta-9-THC on emotional responses to acute psychosocial stress. Drug Alcohol. Depend..

[30] Calabrese EJ, Rubio-Casillas A (2018). Biphasic effects of THC in memory and cognition. Eur. J. Clin. Invest..

[31] Nidadavolu P (2021). Efficacy of Δ9 -tetrahydrocannabinol (THC) alone or in combination with a 1:1 ratio of cannabidiol (CBD) in reversing the spatial learning deficits in old mice. Front. Aging Neurosci..

[32] Sarne Y, Toledano R, Rachmany L, Sasson E, Doron R (2018). Reversal of age-related cognitive impairments in mice by an extremely low dose of tetrahydrocannabinol. Neurobiol. Aging.

[33] Snigdha S (2016). H3K9me3 inhibition improves memory, promotes spine formation, and increases BDNF levels in the aged hippocampus. J. Neurosci..

[34] Tanaka J (2008). Protein synthesis and neurotrophin-dependent structural plasticity of single dendritic spines. Science.

[35] Koleske AJ (2013). Molecular mechanisms of dendrite stability. Nat. Rev. Neurosci..

[36] Njoo C, Agarwal N, Lutz B, Kuner R (2015). The cannabinoid receptor CB1 interacts with the WAVE1 complex and plays a role in actin dynamics and structural plasticity in neurons. PLoS Biol..

[37] Feng G, Mellor RH, Bernstein M, Keller-Peck C, Nguyen QT, Wallace M, Nerbonne JM, Lichtman JW, Sanes JR (2000). Imaging neuronal subsets in transgenic mice expressing multiple spectral variants of GFP. Neuron.

[38] Pologruto TA, Sabatini BL, Svoboda K (2003). ScanImage: Flexible software for operating laser scanning microscopes. Biomed. Eng. Online.

