# Supplementary material for: Chronic low-dose Δ9-tetrahydrocannabinol (THC) treatment stabilizes dendritic spines in 18-month-old mice
Source: Sci Rep. 2023 Jan 25;13:1390. doi: 10.1038/s41598-022-27146-2 (PMC9877016; doi:10.1038/s41598-022-27146-2)
Supplement: Supplementary file 1 — Supplementary Information. [file 41598_2022_27146_MOESM1_ESM.docx]

Supplementary Material

Chronic low-dose Δ^9^ -tetrahydrocannabinol (THC) treatment stabilizes dendritic spines in 18-month-old mice

Joanna Agnieszka Komorowska-Müller^1, +^, Anne-Kathrin Gellner^3,2, +^, Kishore Aravind Ravichandran^2,1^, Andras Bilkei-Gorzo^1^, Andreas Zimmer^1^*, Valentin Stein^2^*

^1^ Institute of Molecular Psychiatry, Medical Faculty, University of Bonn, Bonn, Germany

^2^ Institute of Physiology II, Medical Faculty, University of Bonn, Bonn, Germany

^3^ Department of Psychiatry and Psychotherapy, University Hospital Bonn, Bonn, Germany

+These authors have contributed equally to this work and share first authorship

*** Correspondence:**Valentin Stein
vstein@uni-bonn.de

Andreas Zimmer
a.zimmer@uni-bonn.de

**
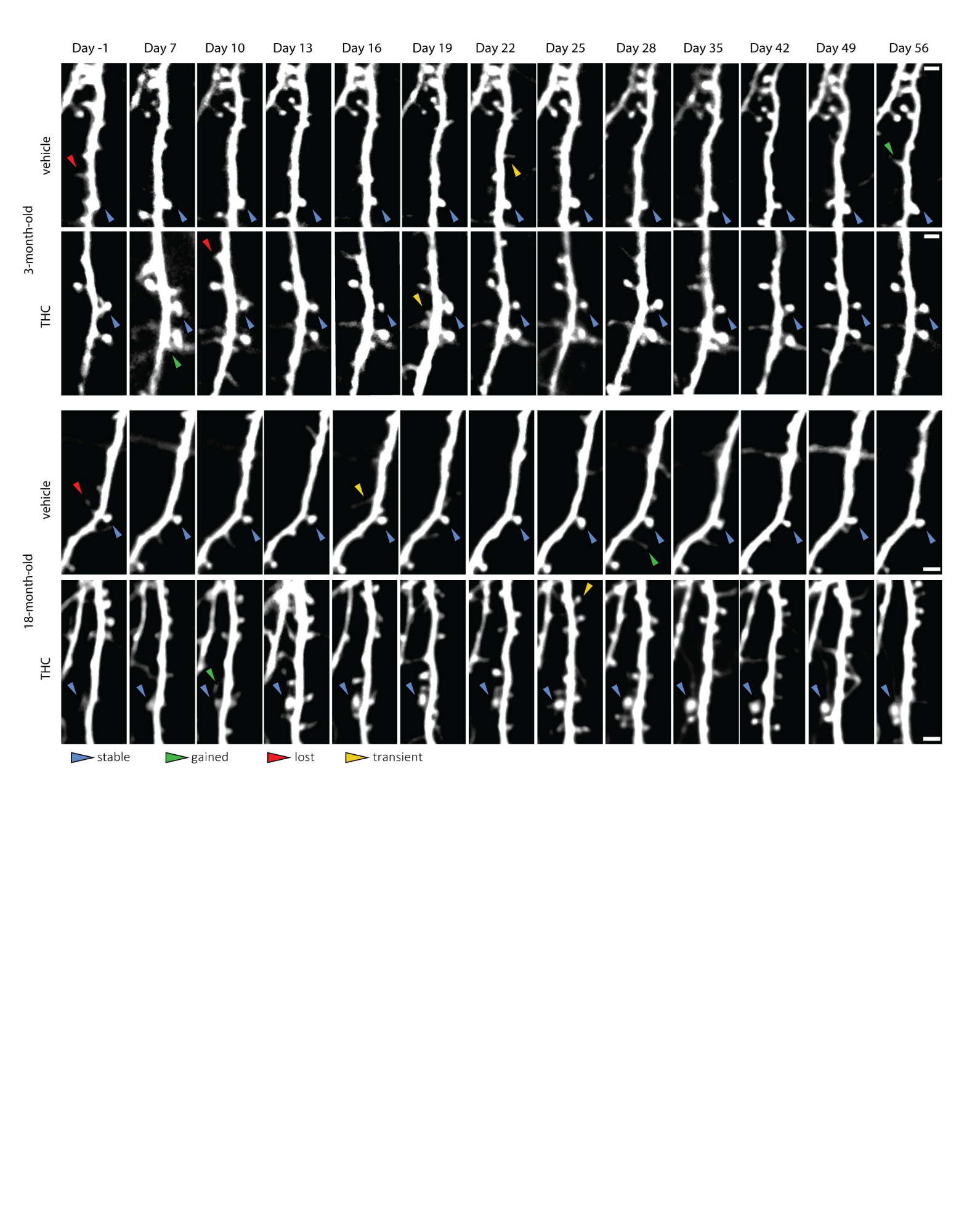
**

**Extended Fig. 1.** Representative images of the same dendritic segment acquired during in vivo imaging from 3- and 18-month-old mice treated with vehicle or THC. The same dendritic segment was imaged before (day -1), during (day 7 to day 28) and after (day 35 to 56) the treatment with either vehicle or THC. Example dynamic spines are indicated with arrows. Scale bar is 2 μm.


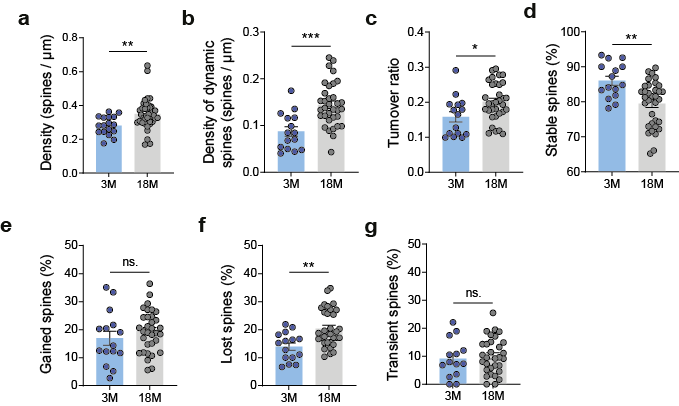


**Extended Fig. 2.** Spine dynamics during baseline imaging (day -4 to -1) reveal age-related changes. (**a**) Density of spines. (**b**) Density of dynamic (lost and gained) spines. (**c**) Turnover ratio - number of lost and gained spines between two consecutive timepoints divided by the total number of spines in both timepoints. Percentage of spines that were stable (**d**), gained (**e**), lost (**f**) between two consecutive timepoints and transient (**g**) i.e. gained in one timepoint and lost in the consecutive one. 3M: 3-month-old mice (n = 14-17 ROIs, N = 8 mice); 18M: 18-month-old mice (n = 31-39 ROIs, N = 18 mice). (**a**) density of spines: U = 154, p = 0.0017; (**b**) density of dynamic spines: t_47_ = 4.101, p = 0.0002; (**c**) turnover ratio: t_46_ = 2.568, p = 0.0135; (**d**) stable spines: U = 114.5, p = 0.0018; (**e**) gained spines: t_46_ = 1.000; p = 0.3223; (**f**) lost spines: U = 114.5, p = 0.0025; (**g**) transient spines: t_43_ = 0.5035, p = 0.6172. Each datapoint represents one ROI. Bars indicate mean with SEM; data was analyzed using unpaired T-test/U-test; * P < 0.05, ** P < 0.01; *** P < 0.001.

**Supplementary Table 1.** Number of analyzed regions of interest (ROIs) in each timepoint. In brackets number of mice.

| **Age** | **Group** | **day -7** | **day -4** | **day -1** | **day 7** | **day 10** | **day 13** | **day 16** | **day 19** | **day 22** | **day 25** | **day 28** | **day 35** | **day 42** | **day 48** | **day 56** |
| --- | --- | --- | --- | --- | --- | --- | --- | --- | --- | --- | --- | --- | --- | --- | --- | --- |
| **3-month-old** | **vehicle** | 5  (3) | 10 (4) | 9  (4) | 10 (4) | 10 (4) | 10 (4) | 7  (3) | 7  (3) | 7  (3) | 7  (3) | 6  (2) | 6  (2) | 7  (3) | 6  (2) | 5  (2) |
|  | **THC** | 9  (3) | 7  (3) | 8  (4) | 10 (4) | 10 (4) | 11 (4) | 11 (4) | 10 (4) | 11 (4) | 10 (4) | 10 (4) | 10 (4) | 11 (4) | 12 (4) | 7  (3) |
| **18-month-old** | **vehicle** | 15 (8) | 12 (7) | 16 (8) | 15 (8) | 15 (7) | 15 (8) | 13 (7) | 13 (7) | 14 (7) | 13 (7) | 12 (6) | 11 (6) | 9  (4) | 8  (4) | 9  (4) |
|  | **THC** | 17 (8) | 21 (10) | 22 (10) | 20 (9) | 17 (9) | 20 (10) | 22 (10) | 20 (9) | 21 (10) | 21 (10) | 19 (9) | 16 (8) | 18 (8) | 12 (7) | 8  (5) |

**Supplementary Table 2.** Detailed statistics table. For post hoc tests, only significant effects are reported. Ns. – not significant.

|  | | **Statistical test** | | **Post hoc test** | |
| --- | --- | --- | --- | --- | --- |
| **Figure** | **Panel** | **Test name** | **Result** | **Test name** | **Result** |
| **1. Long-term low-dose THC-treatment increases spine density in old, but not in young mice.** | **3b:** 3-month-old | 2-way ANOVA (Mixed-effects model (REML)) | **time effect** F_14,192_ = 1.518, p = 0.1074; **treatment effect** F_1,18_ = 0.6493, p = 0.4312; **interaction effect** F_14,192_ = 3.247, p = 0.0001 | Šídák's multiple comparisons test | ns. |
|  | **3c:** 18-month-old | 2-way ANOVA (Mixed-effects model (REML)) | **time effect** F_14,396_ = 0.7745, p = 0.6971; **treatment effect** F_1,37_ = 13.04, p = 0.0009; **interaction effect** F_14,396_ = 5.069, p < 0.0001 | Šídák's multiple comparisons test | **Treatment effect:**  **day 25** p = 0.0141; **day 35** p = 0.0036; **day 42** p = 0.0035; **day 48** p < 0.0001; **day 56** p < 0.0001  **Time effect in comparison to day -1:**  **vehicle: day 56** p = 0.0322  **THC: day** 35 p = 0.0093, **day** 42 p = 0.0008, **d49** p = 0.0064, **d56** p = 0.0007 |
| **2. Long-term low-dose THC-treatment differently alters spine dynamics in old and young mice.**  **2. Long-term low-dose THC-treatment differently alters spine dynamics in old and young mice.** | **2a:** turnover ratio (3-month-old) | 2-way ANOVA (Mixed-effects model (REML)) | **time effect** F_13,179_ = 3.718, p < 0.0001; **treatment effect** F_1,20_ = 10.93, p = 0.0035; **time effect** F_13,179_ = 1.605, p = 0.0872 | Šídák's multiple comparisons test | **day 7** p = 0.0017; **day 10** p = 0.0033; **day 13** p = 0.0014 |
|  | **2b:** gained spines (3-month-old) | 2-way ANOVA (Mixed-effects model (REML)) | **time effect** F_13,179_ = 2.291, p = 0.0080; **treatment effect** F_1,20_ = 9.589, p = 0.0057; **interaction effect** F_13,179_ = 3.050, p = 0.0004 | Šídák's multiple comparisons test | **day 7** p < 0.0001; **day 35** p = 0.0031 |
|  | **2c:** lost spines (3-month-old) | 2-way ANOVA (Mixed-effects model (REML)) | **time effect** F_13,179_ = 3.245, p = 0.0002; **treatment effect** F_1,20_ = 10.15, p = 0.0046; **interaction effect** F_13,179_ = 2.382, p = 0.0057 | Šídák's multiple comparisons test | **day 10** p = 0.0054; **day 13** p = 0.0011 |
|  | **2d:** transient spines (3-month-old) | 2-way ANOVA (Mixed-effects model (REML)) | **time effect** F_9,117_ = 3.715, p = 0.0004; **treatment effect** F_1,20_ = 9.299, p = 0.0063; **interaction effect** F_9,117_ = 2.812, p = 0.0050 | Šídák's multiple comparisons test | **day 7** p = 0.0002; **day 10** p = 0.0237 |
|  | **2e:** survival probability of spines (3-month-old) | log rank test (Mantel-Cox test) | **survival gained spines:** p = 0.9834; **survival all spines:** p = 0.0002 |  |  |
|  | **2f:** turnover ratio (18-month-old) | 2-way ANOVA (Mixed-effects model (REML)) | **time effect** F_13,339_ = 4.759, p < 0.0001; **treatment effect** F_1,36_ = 5.403, p = 0.0259; **interaction effect** F_13,339_ = 1.336, p = 0.1899 | Šídák's multiple comparisons test | **day 16** p = 0.0424; **day 25** p = 0.0448 |
|  | **2g**: gained spines (18-month-old) | 2-way ANOVA (Mixed-effects model (REML)) | **time effect** F_13,339_ = 3.208, p = 0.0001; **treatment effect** F_1,36_ = 3.178, p = 0.0831; **interaction effect** F_13,339_ = 1.285, p = 0.2198 | Šídák's multiple comparisons test | ns. |
|  | **2h:** lost spines (18-month-old) | 2-way ANOVA (Mixed-effects model (REML)) | **time effect** F_13,339_ = 2.356, p = 0.0050; **treatment effect** F_1,36_ = 7.416, p = 0.0099; **interaction effect** F_13,339_ = 1.061, p = 0.3924 | Šídák's multiple comparisons test | ns. |
|  | **2i:** transient spines (18-month-old) | 2-way ANOVA (Mixed-effects model (REML)) | **time effect** F_9,239_ = 1.948, p = 0.0462; **treatment effect** F_1,36_ = 8.564, p = 0.0059; **interaction effect** F_13,339_ = 1.274, p = 0.2518 | Šídák's multiple comparisons test | **day 10** p = 0.0343 |
|  | **2j:** survival probability of spines (18-month-old) | log rank test (Mantel-Cox test) | **survival gained spines:** p < 0.0001; **survival all spines:** p = 0.0123 |  |  |
| **3.** **THC counteracts the effects of aging on the level of spine dynamics.** | **3a:** lost spines | one-way ANOVA / unpaired t-test | **group effect** F_2,36_ = 4.755, p = 0.0147 (3- vs 18-month-old vehicle p = 0.0129) / **18-month-old vehicle vs. THC** t_30_ = 2.321, p = 0.0273 | Dunnett’s multiple comparison test / - | **3- vs 18-month-old vehicle** p = 0.0129 |
|  | **3b:** gained spines | one-way ANOVA / unpaired t-test | **group effect** F_2,36_ = 2.244, p = 0.1207 / ns. | Dunnett’s multiple comparison test / - | ns. |
|  | **3c:** turnover ratio | one-way ANOVA / unpaired t-test | **group effect** F_2,36_ = 3.462, p = 0.0422 / **18-month-old vehicle vs THC** t_30_ = 2.241, p = 0.0326 | Dunnett’s multiple comparison test / - | **3- vs 18-month-old vehicle** p = 0.0644 |
|  | **3d:** stable spines | one-way ANOVA / unpaired t-test | **group effect** F_2,36_ = 4.755, p = 0.0147 / **18-month-old vehicle vs THC** t_30_ = 2.321, p = 0.0273 | Dunnett’s multiple comparison test / - | **3- vs 18-month-old vehicle** p = 0.0129 |
|  | **3e:** survival probability of spines | log rank test (Mantel-Cox test) | **all spines 3- vs 18-months-old vehicle group:** p = 0.0023**; 3-month-old vehicle group vs 18-month old THC group:** p = 0.0076 |  |  |
